# Low-Dose Naltrexone for Neuropathic Pain: Mechanistic Plausibility, Phenotype Transferability, and the Limits of Current Clinical Evidence

**DOI:** 10.3390/life16101650

**Published:** 2026-09-30

**Authors:** Yoo Jung Park, Jihyun Jeon, Sung Jun Kim, Ho Sik Moon, Siwook Chung

**Affiliations:** 1 Department of Anesthesiology and Pain Medicine, St. Vincent’s Hospital, College of Medicine, The Catholic University of Korea, Suwon 16247, Republic of Korea; pyj@catholic.ac.kr; 2 Department of Anesthesiology and Pain Medicine, Eunpyeong St. Mary’s Hospital, College of Medicine, The Catholic University of Korea, Seoul 03312, Republic of Korea; 79chinni@naver.com (J.J.); zero0s@naver.com (S.J.K.); mhsjshcat@catholic.ac.kr (H.S.M.)

**Keywords:** low-dose naltrexone, neuropathic pain, painful diabetic neuropathy, toll-like receptor 4, microglia, nociplastic pain, phenotype, assay sensitivity, evidence appraisal

## Abstract

Low-dose naltrexone (LDN), commonly 1–6 mg/day, is increasingly used off-label for chronic pain, although evidence specific to neuropathic pain remains uncertain. We conducted a structured narrative search of PubMed/MEDLINE, CENTRAL, and ClinicalTrials.gov through 31 July 2026 and classified clinical reports by study design and reported phenotype ascertainment. No placebo-controlled randomized trial was identified in a clinically characterized neuropathic population. One randomized, double-blind, active-control crossover trial in painful diabetic neuropathy, in which both drugs were titrated by early pain response and tolerability, reported an LDN–amitriptyline difference of 1.50 mm on a 0–100 mm visual analog scale (95% confidence interval −1.11 to 4.13), with fewer short-term adverse events under LDN. Interpretation is limited by questions about the reported within-group variance and the crossover analysis, by uncertain adequacy of the treatment and washout durations, and by the absence of placebo or a prespecified equivalence margin. Placebo-controlled evidence from mixed or nociplastic populations shows no consistent clinically meaningful benefit, and its transferability to lesion-based neuropathic pain has not been demonstrated. The TLR4–microglial rationale is plausible, but measured plasma concentrations at 4.5 mg/day fall several orders of magnitude below those associated with TLR4 inhibition in vitro, whereas μ-opioid receptor occupancy is demonstrable within the LDN dose range. Current evidence supports mechanistic plausibility, not established efficacy.

## 1. Introduction

Neuropathic pain remains difficult to treat. Contemporary evidence syntheses show that recommended pharmacotherapies provide clinically meaningful benefit for only a subset of patients, while adverse effects and comorbidity frequently limit treatment persistence [1]. This therapeutic gap creates legitimate interest in alternatives, but it also increases the risk that biological plausibility, uncontrolled improvement, and evidence from adjacent pain phenotypes are combined into a stronger impression than the underlying data support.

Neuropathic pain results from a lesion or disease affecting the somatosensory nervous system, as in painful diabetic neuropathy. NeuPSIG grading considers the relevant history, neuroanatomically plausible pain distribution, sensory signs, and confirmatory testing [2]. Nociplastic pain refers to altered nociception when the pain is not adequately explained by nociceptive mechanisms related to tissue damage or by a somatosensory lesion or disease. Clinical assessment includes features such as regional or widespread pain hypersensitivity; fibromyalgia is a representative predominantly nociplastic condition [3,4].

Mixed pain is used here to describe coexisting contributions from two or more of these mechanisms, including nociceptive pain arising from activation of nociceptors in non-neural tissues. For example, nociceptive axial low-back pain may coexist with neuropathic leg pain due to a nerve-root lesion [2,5]. “Mixed” describes overlapping contributions rather than a separate mechanism. These descriptors are not mutually exclusive, and a nociplastic component may coexist with nociceptive or neuropathic pain [4].

LDN has attracted increasing off-label use because it is inexpensive, usually well tolerated, and supported by several candidate mechanisms [6,7,8]. Interest specifically in neuropathic pain is linked to experimental evidence that spinal microglial activation contributes to pain hypersensitivity after peripheral nerve injury [9]. Preclinical studies of naltrexone and its stereoisomers suggest modulation of toll-like receptor 4 (TLR4)-related signaling, providing a possible pharmacological link to these neuroimmune processes [10,11]. Early clinical reports in painful diabetic neuropathy [12] and neuropathic corneal pain [13] have further encouraged investigation, although these findings provide a rationale for clinical testing rather than proof of efficacy or human target engagement. Gouda et al. reviewed LDN across pain and non-pain indications and concluded that favorable uncontrolled findings were insufficiently corroborated by placebo-controlled trials to justify routine use [6]. McKenzie et al. characterized chronic pain evidence as preliminary and condition-specific and recommended phenotype-stratified trials [7]. The present review shares these cautions but focuses on the inferences permitted for neuropathic pain. For example, both reviews summarized the diabetic neuropathy trial as showing pain reduction comparable to amitriptyline [6,7]. We distinguish that nonsignificant active-treatment contrast from equivalence or placebo-adjusted efficacy and examine response-guided titration, reported precision, and treatment and washout durations [14,15,16]. We also assess reported phenotype ascertainment separately from study design and compare experimental TLR4-related inhibitory concentrations with measured human plasma exposure [11,17,18]. The contribution is therefore a focused appraisal of mechanistic attribution and cross-phenotype transfer, rather than a claim that earlier reviews overlooked heterogeneity.

This review therefore has three aims. First, we examine the proposed mechanisms of LDN and identify the translational steps that remain unverified in humans. Second, we use a heuristic two-dimensional map to display study design and reported phenotype ascertainment separately. Third, we critically examine what the clinical reports can and cannot support for neuropathic pain. The purpose is not to prove that LDN is ineffective, but to define the inference that is currently defensible and the study designs required to move beyond it.

## 2. Mechanistic Rationale and Translational Uncertainty

### 2.1. Candidate Mechanisms

At the conventional 50 mg dose, naltrexone is a competitive antagonist at μ-, κ-, and δ-opioid receptors. In this review, LDN refers primarily to oral regimens of approximately 1–6 mg/day. This is a literature-informed operational range rather than a validated analgesic therapeutic window; the rationale for this scope is provided in Section 3.1. Three non-mutually exclusive mechanisms are commonly proposed. First, transient opioid-receptor blockade may trigger compensatory changes in endogenous opioid signaling. Whether these changes explain pain relief with LDN remains uncertain [8]. Second, naltrexone or its stereoisomers can modify TLR4/MD-2 signaling in microglia and other immune cells, potentially reducing inflammatory signaling that amplifies pain [10,11]. Third, intermittent blockade of the opioid growth factor–opioid growth factor receptor (OGF–OGFr) pathway has been investigated in cell-proliferation models [19]. However, its contribution to analgesia remains uncertain. These are proposed explanations for a possible analgesic effect, not a proven sequence leading to pain relief. Section 2.2 examines the exposure and target-engagement evidence needed to connect these hypotheses to clinical LDN use.

The TLR4–microglial hypothesis is especially attractive in neuropathic pain because peripheral nerve injury can induce spinal microglial activation and signaling changes that contribute to tactile allodynia in animal models [9,20]. Naltrexone and naloxone reversed neuropathic hypersensitivity non-stereoselectively in preclinical experiments, and opioid-inactive (+)-naltrexone and (+)-naloxone inhibit selected TLR4 downstream pathways in cellular systems [10,11]. These findings establish a plausible experimental route from nerve injury to a pharmacologic target; they do not establish that oral clinical LDN engages that target in humans.

### 2.2. The Translational Gap in the TLR4 Hypothesis

Marketed naltrexone is the opioid-receptor-active (−)-enantiomer, not a racemate. The opioid-inactive (+)-enantiomer helps probe opioid-receptor-independent actions, but opioid inactivity does not establish TLR4 selectivity [10,11,21]. Both enantiomers showed similar inhibition of lipopolysaccharide (LPS)-induced nitric oxide production in a microglial assay [11]. Racemic or stereochemically incompletely characterized preparations cannot resolve enantiomer-specific contributions and should not be treated as interchangeable with either isolated enantiomer. Clinical improvement with (−)-naltrexone does not distinguish opioid-mediated from candidate non-opioid effects [14,22,23].

In an LPS-stimulated BV-2 microglial assay, (+)-naltrexone inhibited nitric oxide production with a half-maximal inhibitory concentration (IC_50_) of 105.5 μM [11]. A human TLR4 reporter-cell study reported inhibition of LPS-induced signaling at nominal concentrations of 30–1000 μM using naltrexone described by the investigators as racemic [18]. These functional findings are assay-specific, not human TLR4 occupancy thresholds.

Naltrexone undergoes substantial first-pass metabolism; conventional-dose information reports 5–40% oral bioavailability and mean elimination half-lives of approximately 4 h for naltrexone and 13 h for 6β-naltrexol. Dose proportionality established at 50–200 mg cannot be assumed at 1–6 mg/day [21]. After 21 days of oral naltrexone 4.5 mg/day in a fibromyalgia trial, median plasma concentrations sampled 1–3 h after dosing were 0.33 μg/L for naltrexone (approximately 0.97 nM) and 5.29 μg/L for 6β-naltrexol [17]. These measurements did not establish the maximum plasma concentration (Cmax). In healthy volunteers, [^11^C]carfentanil positron emission tomography (PET) demonstrated μ-opioid receptor occupancy after single oral doses of 2–50 mg; the apparent dose for 50% occupancy was 5.60 mg (95% CI 3.65–7.54), estimated from scans obtained <8 h after dosing [24]. The exposure–occupancy relationship was time-dependent and does not establish an analgesic dose.

Measured parent-drug plasma exposure was several orders of magnitude below the nominal concentrations associated with inhibition in these assays. However, neither total plasma nor nominal culture concentrations establish unbound CNS target-site exposure, and interpreting 6β-naltrexol requires metabolite-specific potency and exposure data. The comparison therefore does not define a human CNS inhibitory threshold. Human μ-opioid receptor occupancy is demonstrated under the studied single-dose conditions, whereas central TLR4/MD-2 engagement at clinical LDN exposure remains unestablished; circulating cytokine changes are not validated measures of that engagement [22,23]. Available data do not establish a validated relationship linking repeated oral LDN dosing, CNS TLR4/MD-2 engagement, neuroimmune modulation, and neuropathic analgesia. The commonly used 4.5 mg/day regimen remains empirical rather than selected through a validated analgesic exposure–target relationship.

### 2.3. Phenotype Transferability Is an Empirical Question

Neuropathic and nociplastic pain may share sensitization and neuroimmune signaling, while differing in the contribution, location, and timing of those processes [3,4,20]. Peripheral nerve-injury models support lesion-related neuroimmune mechanisms, but do not establish that the same drug-responsive pathway operates across clinical conditions [9,20,25]. Mechanisms may also coexist within an individual, so diagnostic categories should not be treated as biologically isolated groups [2,3,4]. Transferability of an LDN effect is therefore an empirical question: shared terminology alone cannot establish equivalent target engagement or therapeutic response.

**Figure 1:**
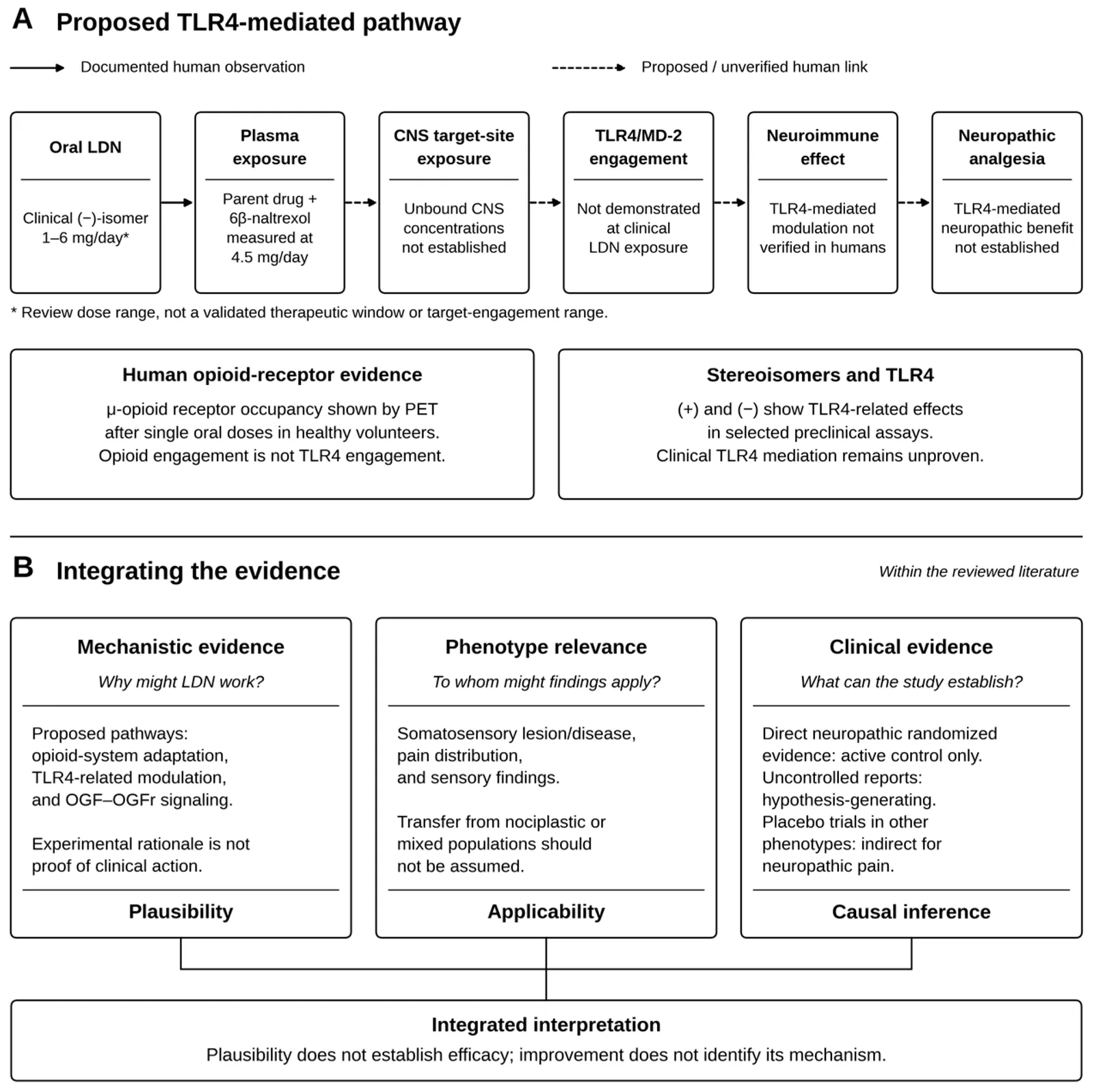
Integrating mechanistic evidence, phenotype relevance, and clinical evidence for LDN. (**A**) The proposed TLR4-mediated exposure-to-effect pathway. The solid arrow denotes documented oral-to-plasma exposure at 4.5 mg/day [17]; dashed arrows indicate links not validated for the human TLR4-mediated pathway. Human μ-opioid receptor occupancy after single oral doses [24] and TLR4-related findings from selected preclinical stereoisomer studies [10,11] are shown separately; neither establishes TLR4-mediated clinical analgesia. The 1–6 mg/day interval denotes the review’s dosing scope, not a validated therapeutic window. (**B**) Mechanistic plausibility, phenotype applicability, and clinical causal inference are complementary domains. Connecting lines indicate integration for interpretation, not a biological sequence or quantitative weighting. CNS, central nervous system; LDN, low-dose naltrexone; MD-2, myeloid differentiation factor 2; OGF–OGFr, opioid growth factor–opioid growth factor receptor; PET, positron emission tomography; TLR4, toll-like receptor 4.

**Figure 2:**
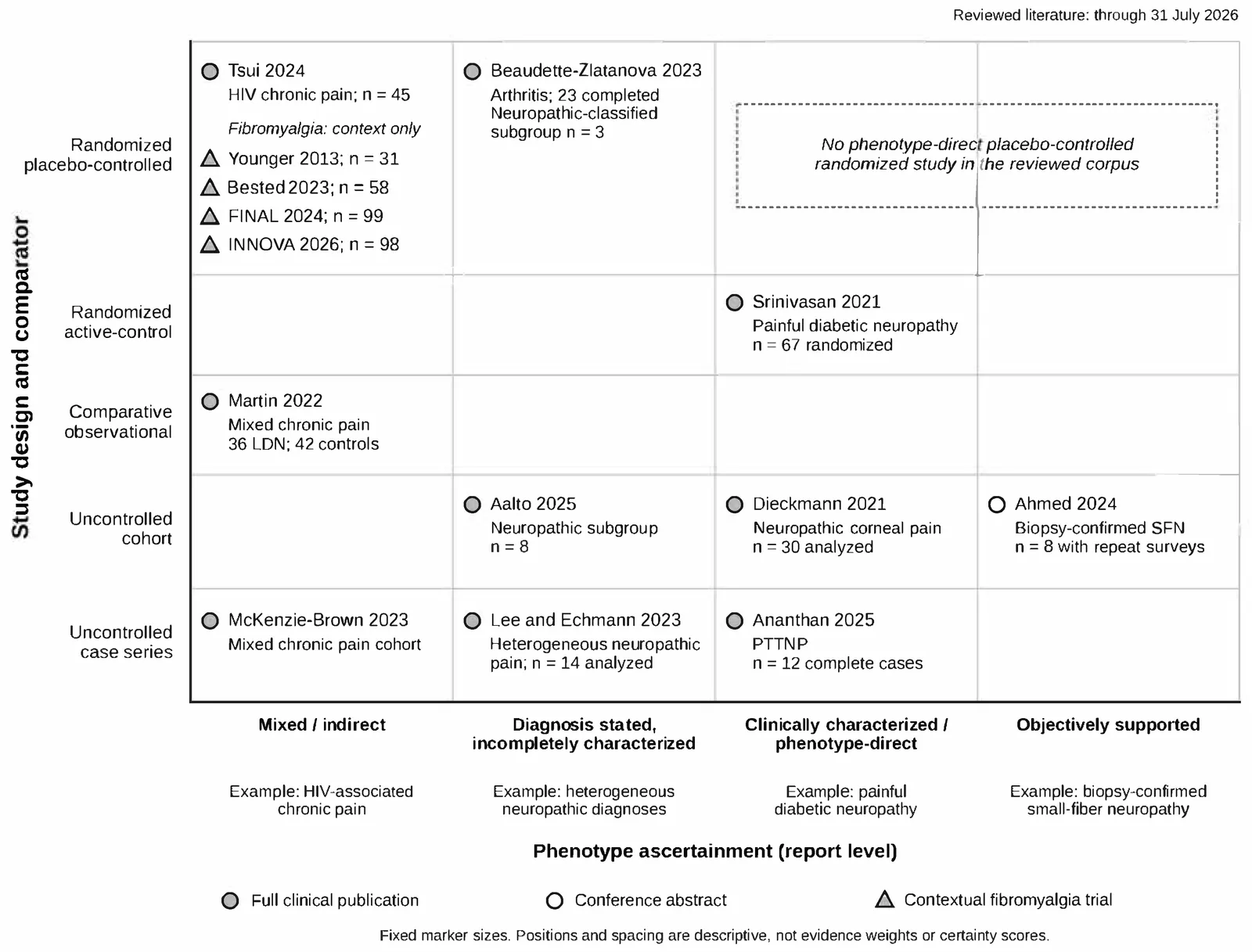
Heuristic map of study design and phenotype ascertainment. The displayed studies are reported in references [13,14,17,23,26,27,28,29,30,31,32,33,34,35]. Filled circles denote full clinical publications, the open circle a conference abstract, and triangles contextual fibromyalgia trials. Categories and examples describe report-level phenotype ascertainment, not individual NeuPSIG grades. Marker sizes are fixed; sample sizes appear only in labels. Neither spacing nor within-category placement carries quantitative weight. This heuristic display is not a validated risk-of-bias or certainty-of-evidence assessment. HIV, human immunodeficiency virus; LDN, low-dose naltrexone; NeuPSIG, Neuropathic Pain Special Interest Group; PTTNP, posttraumatic trigeminal neuropathic pain; SFN, small-fiber neuropathy.

Figure 1 separates mechanistic plausibility, population relevance, and causal inference. Figure 2 provides a heuristic map of reported study design and phenotype ascertainment, which guides the clinical appraisal below.

## 3. Literature Identification and Evidence Classification

### 3.1. Review Design and Information Sources

This was a structured narrative review rather than a systematic review or meta-analysis. PubMed/MEDLINE and the Cochrane Central Register of Controlled Trials (CENTRAL) were searched from database inception through 31 July 2026, and ClinicalTrials.gov was searched on the same date. The electronic strategy combined low-dose naltrexone/naltrexone terms with chronic pain, neuropathic pain, neuropathy, neuralgia, radiculopathy, allodynia, hyperalgesia, small-fiber neuropathy, complex regional pain syndrome, and fibromyalgia terms so that phenotype-indirect placebo-controlled trials relevant to evidence transfer were not missed. No date limits were applied. English-language human reports were considered. Exact search strings and execution dates are provided in Appendix A (Table A1). Reference lists of included reports and recent broad reviews [6,7] were screened, and PubMed related-article links were used as supplementary discovery. No review protocol was registered because the objective was critical narrative synthesis rather than exhaustive certainty grading.

The core clinical synthesis focused on oral naltrexone regimens of approximately 1–6 mg/day in adults with chronic pain when a neuropathic phenotype was stated, clinically separable, or relevant to phenotype transferability. This operational range encompasses initiation at 1–1.5 mg/day in clinical series [26], 2–4 mg/day in the painful diabetic neuropathy trial [14], 4.5 mg/day in corneal pain and fibromyalgia studies [13,27], and 6 mg/day in the FINAL fibromyalgia trial [28]. Fibromyalgia studies inform the discussion of dosing conventions and phenotype transferability, not neuropathic-specific efficacy. The boundaries define the main dosing scope of this review, not a minimum effective dose, a maximum safe dose, or a threshold for TLR4 engagement. Dose-finding evidence extending below 1 mg/day was retained separately as contextual dosing evidence [36]. Real-world reports allowing doses above 6 mg/day were retained only as contextual evidence and were not interpreted as dose-restricted efficacy evidence for the 1–6 mg/day range.

Comparative trials, comparative observational studies, uncontrolled cohorts, case series, and conference abstracts were eligible. Fibromyalgia and other predominantly nociplastic populations were not treated as direct evidence of neuropathic efficacy; placebo-controlled trials in those populations were retained only as contextual evidence about phenotype transfer. Complex regional pain syndrome (CRPS) was treated as mixed/contextual unless CRPS type II or another lesion-based neuropathic phenotype was separately reported. Single-patient case reports were excluded from the evidence map and tabular synthesis; the earlier painful diabetic neuropathy report from the group that later conducted the randomized trial is mentioned only as a historical precursor. Mixed chronic pain reports without separable neuropathic subgroup data were generally excluded from phenotype-specific inference; Martin et al. was retained because it was the only matched comparative observational study identified and therefore informed the study-design dimensions.

Eligibility criteria were applied consistently to the retrieved records. Extracted items included study design, phenotype ascertainment, analyzed or randomized sample, LDN regimen, comparator, treatment duration, pain outcomes, adverse events, and major threats to inference. All included reports and key numerical values used in the manuscript were cross-checked against the source publication or registry record before synthesis. Duplicate independent screening was not performed. Because the purpose was critical interpretation rather than formal certainty grading, validated risk-of-bias instruments and GRADE were not applied. No quantitative synthesis was attempted because only one phenotype-direct randomized comparison was identified and the remaining reports differed substantially in population, design, outcome, and follow-up.

### 3.2. Heuristic Map of Study Design and Phenotype Ascertainment

Figure 2 was constructed as a heuristic map for narrative interpretation rather than a new evidence-grading framework. The vertical dimension describes study design and comparator type, whereas the horizontal dimension describes reported phenotype ascertainment for the plotted cohort or subgroup. These dimensions are presented separately to distinguish design characteristics from population relevance, without assuming statistical independence or assigning a combined quality score. Figure 2 plots reports that directly inform either study-design inference or phenotype transferability. Single-patient reports and studies used only for dose-finding, treatment persistence, or broad prescribing context without separable phenotype-specific outcomes are not plotted; placebo-controlled fibromyalgia trials are shown because they directly inform the phenotype-transfer question.

Four descriptive phenotype categories were used, informed by NeuPSIG diagnostic elements [2]: mixed/indirect populations, illustrated by human immunodeficiency virus (HIV)-associated chronic pain without established neuropathic ascertainment [23]; a diagnosis stated but incompletely characterized, illustrated by heterogeneous neuropathic diagnoses [29]; clinically characterized/phenotype-direct populations, illustrated by painful diabetic neuropathy [14]; and objectively supported neuropathy, illustrated by biopsy-confirmed small-fiber neuropathy [30]. These examples describe the information reported for the relevant cohort or subgroup, not fixed classifications of the underlying diseases.

Placements represent interpretive judgments based on published information rather than formal NeuPSIG grades assigned to individual participants. The “mixed/indirect” category does not imply that mixed pain mechanisms were demonstrated in every participant. Likewise, objective confirmation of neuropathy supports phenotype characterization but does not establish treatment efficacy. Independent duplicate classification and inter-rater reproducibility assessment were not performed, and alternative placements remain possible.

Clinical interpretations rest on the source findings and their limitations, not on diagram coordinates. The map is therefore a visual aid, not a validated risk-of-bias tool or certainty-of-evidence assessment.

## 4. Comparative Clinical Evidence

### 4.1. Painful Diabetic Neuropathy: Phenotype-Direct Active-Control Trial

The most informative phenotype-direct report is a randomized, double-blind, active-control crossover trial in painful diabetic neuropathy [14]. Ninety-nine patients were screened, 71 met eligibility criteria, four withdrew during a 2-week placebo run-in, and 67 were randomized; 61 completed both treatment periods. LDN was initiated at 2 mg/day and could be increased to 4 mg/day; amitriptyline was initiated at 10 mg/day and could be increased to 25 or 50 mg/day. Up-titration was conditional on a VAS pain reduction of less than 20% at follow-up, with tolerability also considered. After initial titration, the selected dose was maintained for the remainder of the treatment period. Each treatment period lasted 6 weeks and was separated by a 2-week washout. Existing painful diabetic neuropathy medications were withdrawn during the run-in, with paracetamol available as rescue medication. In the original trial, randomization codes were generated using a random-block method with random allocation software (version not reported); medication labeling was performed independently, and study drug dispensing was separated from screening and outcome assessment [14].

The reported clinical characteristics are most consistent with probable neuropathic pain under the NeuPSIG grading approach: participants had diabetes, abnormal Diabetic Neuropathy Symptom and Diabetic Neuropathy Examination scores, and a distal pain distribution compatible with neuropathy [2,14]. This is a provisional appraisal of the published cohort description, not an individual reclassification of all participants; detailed sensory mapping and confirmatory testing were not reported sufficiently to establish definite neuropathic pain. Separately, we describe the population as phenotype-direct because painful diabetic neuropathy was the condition specifically studied.

#### 4.1.1. Reported Treatment Effects and Uncertainty

The authors reported per-protocol mean reductions of 26.7 mm (95% CI 24.8–28.5) with LDN and 25.0 mm (23.2–26.8) with amitriptyline on the 0–100 mm VAS. The reported between-treatment difference was 1.64 mm (95% CI −0.92 to 4.20) per protocol and 1.50 mm (−1.11 to 4.13) by intention-to-treat analysis [14]. These estimates are reproduced as reported rather than as independently reanalyzed results.

The apparent precision warrants qualification. Using the reported per-protocol denominator of 61 per treatment and conventional t-based 95% confidence intervals, approximate back-calculation yields change-score standard deviations (SDs) of 7.2 and 7.0 mm, substantially below the approximately 20 mm SD assumed in the sample-size calculation [14]. This discrepancy merits clarification, although a planning assumption need not match observed dispersion, and within-treatment change-score variability is not interchangeable with the variance of a within-participant treatment contrast. The rounded intervals are approximately compatible with the reported unpaired comparison, but this arithmetic consistency does not validate the underlying participant-level variance or the crossover analysis.

The published summaries do not provide sufficient information to independently validate the precision of a paired crossover treatment estimate. We therefore do not use the reported interval to claim that a 10 mm difference has been reliably excluded. The 10 mm planning target was not a prespecified equivalence or non-inferiority margin, and the nonsignificant active-treatment contrast does not establish equivalence, non-inferiority, or placebo-adjusted efficacy [15,16].

#### 4.1.2. What the Active Comparison Cannot Estimate

Without a placebo arm, the trial cannot isolate drug-specific analgesic efficacy. Parallel improvement in two active-treatment periods is compatible with two effective treatments, but it is also compatible with regression to the mean, expectation, repeated assessment, and recovery from withdrawal of background medication during the run-in. In an active-control study, similarity between treatments can mean that both were effective or that neither expressed its expected effect; in the absence of concurrent placebo, assay sensitivity depends heavily on external evidence and similarity of trial conditions [16].

The achieved amitriptyline doses should be interpreted in the context of response-guided titration. Among completers, 23%, 67%, and 10% received 10, 25, and 50 mg/day, respectively [14]. This distribution partly reflects the protocol’s response and tolerability criteria and is not, by itself, evidence of comparator underdosing. The comparison therefore concerns the tested titration regimens rather than fixed-dose treatment effects. Separately, 49.3% of randomized participants had prior amitriptyline exposure, but the previous dose, duration, adherence, and response were not reported; prior exposure cannot be equated with documented treatment failure [14].

Treatment duration should be interpreted alongside the proposed mechanism. The 6-week periods included initial titration, leaving a shorter interval at the maintained dose for participants requiring escalation [14]. If clinically relevant neuroimmune effects develop gradually, this schedule may incompletely characterize their time course. The investigators reported improvement during the first 2 weeks followed by relatively stable pain scores [14]; however, that trajectory does not identify a drug-specific neuroimmune effect or establish the duration required for its full expression.

Washout adequacy is likewise a pharmacodynamic question, not solely a pharmacokinetic one. Although 2 weeks greatly exceeds the reported plasma elimination half-lives of naltrexone and 6β-naltrexol [21], clearance does not demonstrate reversal of any persisting receptor-related or downstream effects. If such effects occur, residual activity could influence the next period’s baseline or outcomes. Delayed onset and prolonged offset have not been established in this population, so neither insufficient treatment duration nor actual carryover can be asserted; rather, the timing assumptions remain insufficiently validated.

The primary change-score contrast was reported using an unpaired t-test, and additional mixed-effects analyses found no significant period, sequence, or treatment-by-period effects [14]. These null findings do not establish negligible carryover: such tests have limited ability to resolve residual effects in small two-period crossover trials [15]. The unresolved onset and offset of any clinically relevant action therefore remain important constraints on interpretation, and the direction or magnitude of any resulting bias cannot be determined from the published summaries.

#### 4.1.3. Tolerability and Risk of Functional Unblinding

The short-term tolerability contrast is the most directly interpretable result. The report tabulated adverse events during treatment exposure in 8 of 67 participants for LDN and 36 of 67 for amitriptyline, corresponding to 8 versus 52 events (*p* < 0.001) [14]. Daytime somnolence, dry mouth, and postural hypotension occurred predominantly or exclusively with amitriptyline, whereas mild diarrhea was the most characteristic LDN event. Improvement in sleep quality was more frequent with amitriptyline (odds ratio 2.22, 95% CI 1.02–4.93; *p* = 0.04). The marked asymmetry in recognizable anticholinergic and sedative effects makes functional unblinding plausible for a subjective outcome, despite nominal double blinding. The trial supports lower short-term adverse-event burden with LDN than with this amitriptyline regimen; it does not establish long-term safety.

### 4.2. Placebo-Controlled but Phenotype-Indirect Evidence

#### 4.2.1. HIV and Chronic Pain

A three-arm pilot randomized trial assigned 45 people with HIV, chronic pain, and past-year heavy alcohol use to LDN 4.5 mg/day, gabapentin up to 1800 mg/day, or placebo for 8 weeks [23]. The trial is important because it provides placebo anchoring, but the pain population was broad and was not characterized by a somatosensory lesion, neuroanatomically plausible distribution, sensory signs, or confirmatory testing. It is therefore placebo-controlled evidence for chronic pain, not direct evidence for neuropathic pain.

LDN was not superior to placebo for pain severity (adjusted difference 0.88 on a 0–10 scale, 95% CI −0.70 to 2.46) or pain interference (0.40, 95% CI −1.18 to 1.99) [23]. No clear between-group differences were observed in the measured inflammatory biomarkers. The sample was small, CIs were wide, and three participants permanently discontinued LDN; these findings do not provide a precise negative estimate. They nevertheless show why a placebo-controlled result in a mixed population should be interpreted separately from phenotype-direct neuropathic evidence.

#### 4.2.2. Arthritis with painDETECT Classification

A randomized, double-blind, placebo-controlled crossover pilot enrolled adults with osteoarthritis or inflammatory arthritis. Twenty-three participants completed 8 weeks of LDN 4.5 mg/day and 8 weeks of placebo. Among participants with reported painDETECT classification, pain was classified as nociceptive in nine, mixed in eight, and neuropathic in three. Mean change in Brief Pain Inventory pain interference was −23 (standard deviation 19.4) during LDN and −22 (19.2) during placebo (*p* = 0.90), with no significant differences in pain severity, fatigue, depression, or health-related quality of life [31].

This trial provides a second placebo-anchored result in a predominantly nociceptive or mixed population, and it is the only placebo-controlled study identified here in which pain phenotype was characterized prospectively with a screening instrument rather than inferred from the underlying diagnosis. That is also why its limits are instructive. The three participants classified as neuropathic were not reported as a separate outcome subgroup, and the investigators concluded that too few patients had been enrolled either to rule out a modest benefit or to assess neuropathic pain. The caution we apply to the active-control trial applies symmetrically here: a null result in an underpowered pilot is not evidence of absence. The trial therefore informs phenotype transferability without estimating efficacy in a neuropathic condition, and it illustrates how rarely the phenotype at issue has been sampled even in placebo-controlled work [31].

### 4.3. Comparative Observational Evidence

A matched retrospective cohort compared 36 patients treated with LDN for at least 2 months with 42 matched controls in a mixed chronic pain population [32]. The study reported a larger percentage reduction in pain in the LDN group (−37.8% versus −4.3%) and a number needed to treat of 3.2 for a 50% reduction in pain. The investigators also observed that patients with neuropathic pain appeared to benefit more than those with nociceptive or inflammatory pain; this was an unprespecified subgroup observation in a non-randomized cohort and is hypothesis-generating rather than confirmatory. Matching improves comparability on recorded variables but cannot remove confounding by indication, treatment selection, expectation, persistence, concurrent care, or the pain trajectory at the time of prescribing. The parent cohort was not a phenotype-direct neuropathic population, and the neuropathic subgroup was not separately characterized against NeuPSIG criteria. The study therefore supports an association in routine care; neither its number needed to treat nor its neuropathic subgroup signal should be treated as a randomized causal effect or imported into neuropathic pain practice. Table 1 summarizes the principal clinical evidence relevant to LDN and neuropathic pain.

## 5. Uncontrolled Phenotype-Relevant Evidence

Uncontrolled reports show that LDN has been used in clinically defined neuropathic conditions and that some treated patients report improvement. In neuropathic corneal pain, 30 of 59 identified patients were analyzed after at least 4 weeks of LDN, with mean pain decreasing from 6.13 to 3.23 on a 0–10 scale; 16 of 30 reported at least 50% improvement [13]. Most participants received concomitant therapies, and the analyzed sample represented approximately half of those identified. The result is compatible with benefit in selected patients but cannot separate drug effect from natural fluctuation, regression to the mean, co-intervention, or selective follow-up.

In posttraumatic trigeminal neuropathic pain, 21 records met the case-series criteria and 12 complete cases were included in the final analysis. Pain ratings decreased at follow-up, and no significant adverse effects were reported [33]. The International Classification of Orofacial Pain criteria strengthened phenotype definition, but complete-case analysis, concurrent medication, and the absence of a comparator prevent causal attribution. A conference abstract in biopsy-confirmed cryptogenic small-fiber neuropathy identified 44 biopsy-positive patients; 13 initiated LDN and eight had repeat symptom surveys, with improvement across five survey domains [30]. Objective confirmation of small-fiber neuropathy strengthens phenotype characterization, but an eight-person complete-case analysis reported only in abstract form cannot estimate treatment effect.

A heterogeneous retrospective series reported improvement in 14 analyzed patients with persistent pain treated with 1.5–4.5 mg/day of LDN [29]. Because diagnoses, co-interventions, and follow-up varied, this report is best treated as mixed evidence rather than confirmation in a single neuropathic phenotype. The earlier single-patient report by Hota et al. [12] is mentioned solely as a historical precursor, not as part of the evidence map or tabular synthesis and not as support for efficacy or safety conclusions.

Concordant improvement across uncontrolled reports does not resolve these limitations when the reports share the same direction of bias. In off-label care, patients who initiate, persist with, return for follow-up, and have their response documented are unlikely to be a random sample of everyone prescribed the drug. Repetition of a favorable complete-case pattern can therefore create apparent consistency without independent confirmation.

## 6. Mixed Chronic Pain Cohorts and Phenotype Transfer

Mixed chronic pain cohorts are informative about prescribing, adherence, tolerability, and possible responder heterogeneity, but their headline response rates should not be applied directly to neuropathic pain. In one academic pain-center series, 44% of 137 patients prescribed LDN had no evidence of filling the prescription. Among patients with charted responses, those reporting more than 50% pain relief were more likely to carry neuropathic pain or complex regional pain syndrome diagnoses [26]. Because non-initiation was substantial, outcomes were unblinded and retrospective, and CRPS is mechanistically mixed, the association is hypothesis-generating.

In a later real-world cohort, 93 of 128 prescribed patients met inclusion criteria and initiated LDN; 53.8% were judged to have subjective benefit overall. Four of eight patients in the neuropathic pain subgroup and one of three patients with CRPS were reported to benefit [34]. The neuropathic result is therefore a description of four patients, not a stable subgroup estimate. Other real-world studies demonstrate substantial variability in effective dose and treatment persistence [36,37]. These data support individualized dose-finding as a practical reality, but they do not define an evidence-based optimal dose or identify a validated responder phenotype.

Fibromyalgia illustrates why phenotype and design should remain explicit. A small randomized crossover trial in 31 women generated much of the early enthusiasm for LDN [27], but later placebo-controlled trials have not shown a consistent population-average analgesic benefit. In a randomized, double-blind crossover trial of 58 patients, the two primary outcomes did not favor LDN and the investigators concluded that the data did not indicate clinically relevant analgesic efficacy [17]. In the 99-participant FINAL parallel-group trial, 12 weeks of naltrexone 6 mg/day was not superior to placebo for pain intensity (between-group difference −0.34 on a 0–10 scale, 95% CI −0.95 to 0.27) [28]. The 2026 INNOVA trial randomized 98 women to LDN 4.5 mg/day or placebo and followed outcomes for 12 months; at 3 months, the adjusted between-group difference in pain change was 0.49 points in favor of placebo (95% CI −0.32 to 1.31; *p* = 0.236; d = 0.19), and no clinically meaningful advantage emerged at 6 or 12 months [35]. Some meta-analyses have nevertheless reported favorable pooled estimates, particularly in fibromyalgia [38]. Those syntheses combine a small and heterogeneous trial set and do not address lesion-based neuropathic pain; a subsequent re-analysis of a separate fibromyalgia meta-analysis reported lower efficacy than originally estimated [39]. Taken together, the fibromyalgia literature neither proves universal ineffectiveness nor supports transfer of efficacy to neuropathic pain. It instead demonstrates why placebo anchoring, adequate sample size, and phenotype-specific inference matter.

## 7. Safety, Dosing, and Practical Considerations

Across the pain studies reviewed, reported adverse effects were generally mild and included gastrointestinal symptoms, vivid dreams, headache, and sleep disturbance [6,7,13,14,17,23,26,27,28,29,30,31,32,33,34,35]. The painful diabetic neuropathy trial provides the clearest phenotype-direct participant-level comparison and showed fewer short-term adverse events with LDN than with amitriptyline [14]. That pattern was not uniform across placebo-controlled studies, however. In the three-arm HIV pilot, adverse events were more frequent in the LDN arm (13 events) than in the placebo arm (5 events), three participants permanently discontinued LDN, and the blind was broken for one LDN participant with a serious adverse event requiring medical attention [23]. A favorable tolerability profile relative to a sedating comparator therefore does not establish a favorable profile relative to placebo. The INNOVA fibromyalgia trial extends randomized exposure to 12 months in 98 participants and found similar overall adverse-event proportions with LDN and placebo, with no treatment-related serious adverse events [35]. This longer phenotype-indirect study is reassuring but remains far too small to characterize uncommon, reproductive, hepatic, or multi-year risks in neuropathic populations. “Generally well tolerated in available studies” should therefore not be converted into “established long-term safety.”

No standardized analgesic dose has been established. Although 4.5 mg/day is frequently studied, observational dose-finding has reported individually effective doses of approximately 0.1–6 mg/day [36]. These uncontrolled observations illustrate dosing variability rather than establish an optimal regimen. The 1–6 mg/day interval used to organize this review should therefore not be interpreted as a recommended prescribing range. Dose, titration interval, time to declare non-response, and maintenance duration are not supported by comparative dose–response trials in neuropathic pain. A clinically reasonable off-label trial should therefore be time-limited, use prespecified continuation criteria, and assess pain intensity together with function, sleep, rescue medication, and patient-defined goals rather than relying on a global impression alone.

Where suitable commercial low-strength formulations are unavailable, LDN may require pharmacy compounding, as described in clinical practice reports [26]. Dose accuracy, formulation consistency, and storage conditions should therefore be considered. Compounded preparations should be obtained from appropriately regulated pharmacies using applicable quality standards; poorly prepared products may contain incorrect amounts of active ingredient [40].

LDN remains pharmacologically relevant to opioid treatment. The approved oral naltrexone label contraindicates use in patients receiving opioid analgesics or with current opioid dependence, warns about precipitated withdrawal, and notes that opioid analgesia may be difficult to provide during receptor blockade [21]. The magnitude and duration of blockade at LDN doses are not defined by the pain literature, and no validated perioperative interruption interval specific to LDN exists. Medication reconciliation should therefore include opioids, including tramadol and opioid-containing antitussive or antidiarrheal products, and anticipated surgery or acute opioid analgesia should be planned in advance rather than assuming that a low dose is clinically inert.

## 8. Ongoing and Registered Studies

ClinicalTrials.gov records rechecked on 31 July 2026 indicate that more direct evidence may emerge. Registered studies include a placebo-controlled trial in painful diabetic neuropathy [41], a study of neuropathic pain in people with HIV [42], a pilot study of central neuropathic pain after traumatic spinal cord injury [43], and a placebo-controlled study in complex regional pain syndrome [44]. The key advance will not be the number of registered studies but whether they use placebo anchoring, credible phenotype ascertainment, prespecified analysis, adequate duration, and participant-level safety reporting. Table A2 in Appendix A summarizes the records reviewed on 31 July 2026.

## 9. Discussion

### 9.1. What the Current Evidence Supports

The phenotype-direct randomized evidence reviewed consists of one painful diabetic neuropathy active-control trial [14]. Its nonsignificant contrast compares response-titrated regimens; the achieved dose distribution does not by itself establish comparator underdosing. Uncertainty about reported precision and the adequacy of treatment and washout durations limits interpretation, as detailed in Section 4.1. Fewer short-term adverse events were reported with LDN, but the trial does not establish equivalence, non-inferiority, or placebo-adjusted efficacy [14,15,16].

The HIV and arthritis placebo-controlled pilots did not demonstrate superiority, but their small samples and incomplete neuropathic ascertainment preclude a definitive neuropathic-specific conclusion [23,31]. Fibromyalgia trials provide contextual evidence with inconsistent population-average benefit, not direct estimates for lesion-based neuropathic pain [17,27,28,35]. Comparative observational and uncontrolled reports remain hypothesis-generating [13,26,29,30,32,33,34,36,37]. Thus, grouping all randomized or favorable reports together cannot resolve the evidence gap: the comparator, study limitations, and population actually studied determine what each report contributes (Figure 1B and Figure 2; Table 2).

### 9.2. Implications for Mechanistic Interpretation

The exposure appraisal in Section 2.2 constrains mechanistic attribution, not the possibility of benefit through other pathways: concentrations associated with TLR4-related inhibition in vitro lie far above those measured during clinical LDN dosing, whereas μ-opioid receptor occupancy is demonstrable within the same oral dose range [11,17,18,24]. A positive clinical trial could establish efficacy without identifying its mechanism; conversely, opioid-receptor occupancy or a circulating cytokine change would not itself establish TLR4-mediated analgesia. Mechanistic claims therefore require target-specific evidence at the tested exposure, separately from evidence that treatment improves clinical outcomes.

### 9.3. What an Answerable Trial Would Require

A trial capable of establishing efficacy in neuropathic pain should enroll a prespecified neuropathic condition and report which NeuPSIG elements are satisfied. Sensory examination should be required, and confirmatory testing should be used where feasible. A concurrent placebo arm is recommended to estimate the treatment effect of LDN directly against placebo under the trial conditions; an active comparator may be added to contextualize efficacy and tolerability. If equivalence or non-inferiority is intended, the margin and analysis populations must be prespecified.

The trial should also specify background medication management, run-in procedures, rescue therapy, outcome direction, clinically meaningful thresholds, and the handling of missing data. Blinding should be assessed because recognizable adverse effects may reveal treatment allocation. Treatment duration should allow assessment after titration and characterization of response over time. If crossover is used, the interval between treatments and the timing of outcome assessment should be justified by the expected duration of effects, rather than plasma half-life alone [15]. Adverse events should be reported as both affected participants and events, with denominators. Mechanistically informative studies should include pharmacokinetics and a validated target-engagement strategy rather than measuring a broad cytokine panel without evidence that the selected markers reflect the relevant pathway.

Until the reversibility and duration of any treatment effect are better characterized, a parallel-group placebo-controlled design would avoid between-period carryover. Replicated N-of-1 trials may subsequently address individual response, but require credible onset and offset assumptions, adequate treatment and washout intervals, and prespecified blinded comparisons; they are not a solution to unresolved carryover [15].

### 9.4. Implications for Practice

An individualized LDN trial may be considered when established therapies are ineffective or unsuitable, provided its off-label status and uncertain benefit are discussed [1]. Before treatment, document the neuropathic diagnosis, baseline pain and function, a patient-defined functional goal, the titration plan, and current or anticipated opioid use [21].

Continuation could require a prespecified benefit, such as at least a 30% reduction from baseline in average pain intensity on a 0–10 numerical rating scale, or a clearly documented improvement in the agreed functional goal, with acceptable tolerability [45]. The 30% pain threshold draws on general chronic pain outcome guidance and has not been validated specifically for LDN. An evidence-based reassessment interval has not been established for LDN in neuropathic pain. Treatment should be stopped earlier for unacceptable adverse effects and discontinued at reassessment if meaningful benefit is absent. Background treatment should remain stable where feasible, and improvement should not be taken as proof of a drug-specific effect.

## 10. Limitations

This review is structured and reproducibly described but remains narrative. We searched PubMed/MEDLINE and CENTRAL and supplemented them with ClinicalTrials.gov, reference-list screening, and PubMed related-article discovery; Embase, Scopus, and Web of Science were not systematically searched, so reports indexed only in those databases may have been missed. English-language restriction, inclusion of conference abstracts, and the decision not to catalog every mixed chronic pain cohort without separable neuropathic data introduce additional selection and reporting limitations. Duplicate independent screening was not performed, validated risk-of-bias instruments were not applied, and certainty was not rated with GRADE. Figure 2 reflects interpretive judgments without independent duplicate classification or an assessment of inter-rater reproducibility. Alternative placements are possible, and the map cannot substitute for formal risk-of-bias or certainty assessment.

No meta-analysis was performed because the clinical studies differed in phenotype and design and only one phenotype-direct randomized comparison was available. Contextual fibromyalgia trials were discussed to test the logic of phenotype transfer, not pooled with neuropathic reports. The mechanistic section is selective rather than a systematic review of neuroimmune biology. Registry records are dynamic and provide no outcome data until results are posted or published. Finally, the interpretation of the painful diabetic neuropathy trial relies on detailed values in the original report; where prior treatment adequacy, confirmatory testing, or analytic details were not reported, we treated them as unknown rather than inferring them from persistent symptoms. The exposure appraisal remains indirect and does not establish human central TLR4 engagement (Section 2.2).

## 11. Conclusions

Searches through 31 July 2026 identified no placebo-controlled randomized trial of LDN in a clinically characterized neuropathic population. The single phenotype-direct trial compared response-titrated LDN with amitriptyline and reported no significant difference and fewer short-term adverse events with LDN, but uncertainties about the reported precision and about the adequacy of the treatment and washout durations limit interpretation, and neither equivalence nor non-inferiority is established. Placebo-controlled evidence comes from mixed or nociplastic populations, and its transfer to lesion-based neuropathic pain has not been demonstrated. The TLR4–microglial rationale remains plausible, but human central TLR4/MD-2 engagement at oral LDN exposure is unestablished, so mechanistic plausibility cannot substitute for phenotype-relevant clinical evidence. What is needed is placebo-controlled trials in prespecified neuropathic conditions, with durations justified by the expected time course of effect and with participant-level safety reporting. Until then, LDN may be an informed, monitored off-label trial after established options are ineffective or unsuitable, not a replacement for evidence-based care.

## Figures and Tables

**Table 1:** Principal clinical evidence relevant to LDN and neuropathic pain.

Study and Design	Population/Phenotype	Intervention/Comparator	Main Outcome and Principal Limitation
Srinivasan et al., 2021 [14] Randomized double-blind active-control crossover	Painful diabetic neuropathy; 67 randomized, 61 completed. Abnormal DNS/DNE; probable neuropathic pain (report-level NeuPSIG appraisal); phenotype-direct.	LDN 2–4 mg/day vs. amitriptyline 10–50 mg/day; response-guided titration; 6 weeks per period with a 2-week washout.	ITT VAS (0–100 mm) difference 1.50 mm (95% CI −1.11 to 4.13); no placebo or prespecified equivalence/non-inferiority margin; fewer short-term adverse events with LDN. Treatment duration and carryover uncertainty.
Tsui et al., 2024 [23] Randomized placebo-controlled pilot, 3 arms	People with HIV, chronic pain, and heavy alcohol use; *n* = 45; neuropathic phenotype not established (mixed/indirect).	LDN 4.5 mg/day vs. gabapentin up to 1800 mg/day vs. placebo; 8 weeks.	No superiority of LDN over placebo for pain severity or interference (0–10 scales); small pilot with wide CIs.
Beaudette-Zlatanova et al., 2023 [31] Randomized double-blind placebo-controlled crossover pilot	Osteoarthritis or inflammatory arthritis; 23 completed; painDETECT: nociceptive *n* = 9, mixed *n* = 8, neuropathic *n* = 3 among those classified.	LDN 4.5 mg/day vs. placebo; 8 weeks per period.	BPI pain-interference score (0–70) change −23 vs. −22 (*p* = 0.90); neuropathic-classified subgroup too small and not reported separately.
Martin et al., 2022 [32] Matched retrospective cohort	Mixed chronic pain; 36 LDN-treated and 42 matched controls; parent cohort not phenotype-direct.	LDN for ≥2 months vs. matched routine-care controls.	Greater pain reduction (−37.8% vs. −4.3%) and reported NNT 3.2 for 50% pain reduction; a neuropathic subgroup appeared to benefit more, but this was an unprespecified observation; observational association rather than a causal or neuropathic-specific estimate.
Dieckmann et al., 2021 [13] Uncontrolled retrospective cohort	Neuropathic corneal pain; 59 identified, 30 analyzed; clinically phenotype-direct.	LDN 4.5 mg nightly for ≥4 weeks; co-interventions common.	Pain 6.13→3.23/10; 16/30 had ≥50% improvement; no comparator and selective follow-up.
Ananthan et al., 2025 [33] Uncontrolled retrospective case series	Posttraumatic trigeminal neuropathic pain meeting ICOP criteria; 21 records, 12 complete cases.	LDN; concurrent medication in some participants.	VAS (0–10) decreased at follow-up; no significant adverse effects reported; no comparator and complete-case analysis.
Ahmed et al., 2024 [30] Conference abstract	Biopsy-confirmed cryptogenic small-fiber neuropathy; 44 diagnosed, 13 initiated LDN, 8 had repeat surveys.	LDN titrated to 4.5 mg/day; variable follow-up.	Five symptom domains decreased among 8 completers; abstract-only, very small complete-case evidence.
Lee and Echmann, 2023 [29] Uncontrolled retrospective series	Heterogeneous persistent neuropathic pain diagnoses; 18 screened, 14 analyzed; phenotype mixed/incompletely characterized.	LDN 1.5–4.5 mg/day; concurrent care common.	NRS (0–10) 6.29→3.71; no comparator, heterogeneous indications, and co-intervention.
McKenzie-Brown et al., 2023 [26]; Aalto et al., 2025 [34] Mixed real-world cohorts	Broad chronic pain cohorts; neuropathic/CRPS subgroup data were small and incompletely separable.	Routine-care LDN; no randomized comparator.	McKenzie-Brown: 44% non-fill; Aalto: 4/8 neuropathic subgroup judged to benefit; descriptive only.

Abbreviations: BPI, Brief Pain Inventory; CI, confidence interval; CRPS, complex regional pain syndrome; DNE, Diabetic Neuropathy Examination; DNS, Diabetic Neuropathy Symptom; **HIV, human immunodeficiency virus**; ICOP, International Classification of Orofacial Pain; ITT, intention-to-treat; LDN, low-dose naltrexone; **NeuPSIG, Neuropathic Pain Special Interest Group**; NNT, number needed to treat; NRS, numerical rating scale; VAS, visual analog scale.

**Table 2:** Permissible inference from the current evidence layers.

Evidence Layer	What It Supports	What It Does Not Support	Most Important Next Step
Phenotype-direct randomized active-control trial [14]	Small reported active-treatment point estimate, with unresolved precision; fewer short-term adverse events reported with LDN.	Drug-specific efficacy; placebo-adjusted effect; formal equivalence/non-inferiority; long-term safety.	Placebo-controlled, adequately analyzed trial in a well-characterized neuropathic condition.
Phenotype-indirect randomized placebo evidence [17,23,27,28,31,35]	Placebo anchoring in mixed/nociplastic populations; no consistent clinically meaningful population-average benefit across the larger recent trials.	A neuropathic-specific efficacy estimate; direct transfer from fibromyalgia or mixed pain to lesion-based neuropathic pain.	Placebo-controlled trial with explicit lesion-based phenotype ascertainment and prespecified analysis.
Comparative observational evidence [32]	Association between treatment and improvement in routine care.	Causal NNT; control of unmeasured confounding; transfer of parent-cohort effect to neuropathic pain.	Prospective randomization or a prespecified comparative cohort with rigorous confounder adjustment.
Uncontrolled phenotype-relevant reports [13,29,30,33]	Feasibility; possible responders; candidate outcomes and safety signals.	Attribution of improvement to LDN; unbiased effect size; comparative safety.	Prospective placebo-controlled study with complete follow-up and stable co-interventions.
Mixed real-world cohorts [26,34,36,37]	Prescribing patterns, initiation, persistence, dose variability, and descriptive subgroup signals.	Phenotype-specific efficacy; validated responder profile; optimal dose.	Prospective phenotype-stratified study and dose-finding linked to outcomes.

Abbreviations: LDN, low-dose naltrexone; NNT, number needed to treat.

## Data Availability

No new datasets were generated for this review. All data discussed are available in the cited publications, registry records, and the appendix to this article.

## Abbreviations

The following abbreviations are used in this manuscript:

BPI	Brief Pain Inventory
CI	Confidence interval
Cmax	Maximum plasma concentration
CNS	Central nervous system
CRPS	Complex regional pain syndrome
DNE	Diabetic Neuropathy Examination
DNS	Diabetic Neuropathy Symptom
GRADE	Grading of Recommendations, Assessment, Development and Evaluations
HIV	Human immunodeficiency virus
IC_50_	Half-maximal inhibitory concentration
ICOP	International Classification of Orofacial Pain
ITT	Intention-to-treat
LDN	Low-dose naltrexone
LPS	Lipopolysaccharide
MD-2	Myeloid differentiation factor 2
NeuPSIG	Neuropathic Pain Special Interest Group
NNT	Number needed to treat
NRS	Numerical rating scale
OGF	Opioid growth factor
OGFr	Opioid growth factor receptor
PET	Positron emission tomography
SD	Standard deviation
TLR4	Toll-like receptor 4
VAS	Visual analog scale

## Appendix A

This appendix reports the full search strategies and the registered studies identified during the evidence horizon scan.

**Table A1:** Search strategies.

Source	Date Searched	Search Strategy
PubMed/MEDLINE	31 July 2026	((“low-dose naltrexone”[Title/Abstract] OR “low dose naltrexone”[Title/Abstract] OR (naltrexone[Title/Abstract] AND (LDN[Title/Abstract] OR “low dose”[Title/Abstract] OR “low-dose”[Title/Abstract]))) AND (pain*[Title/Abstract] OR neuropath*[Title/Abstract] OR neuralgia*[Title/Abstract] OR radiculopath*[Title/Abstract] OR allodynia[Title/Abstract] OR hyperalgesia[Title/Abstract] OR “small fiber neuropathy”[Title/Abstract] OR “small fibre neuropathy”[Title/Abstract] OR “complex regional pain syndrome”[Title/Abstract] OR CRPS[Title/Abstract] OR fibromyalg*[Title/Abstract]))
CENTRAL (Cochrane Library)	31 July 2026	(naltrexone OR “low dose naltrexone” OR “low-dose naltrexone” OR LDN):ti,ab,kw AND (pain* OR analgesi* OR neuralgia* OR neuropath* OR polyneuropath* OR radiculopath* OR hyperalges* OR allodyni* OR CRPS OR “small fiber” OR “small fibre” OR fibromyalg*):ti,ab,kw
ClinicalTrials.gov	31 July 2026	Intervention/other terms: (“low dose naltrexone” OR “low-dose naltrexone” OR LDN); condition/disease terms: (pain OR neuropathy OR neuropathic OR neuralgia OR radiculopathy OR allodynia OR hyperalgesia OR “small fiber neuropathy” OR “small fibre neuropathy” OR “complex regional pain syndrome” OR CRPS OR fibromyalgia). All recruitment statuses were reviewed.
Supplementary discovery	31 July 2026	Reference lists of included reports and the broad reviews by Gouda et al. [6] and McKenzie et al. [7] were screened. PubMed related-article links were checked for additional original reports and publications associated with registry records.

Each search was run from database inception to the date shown. No date filters were applied. The full strategy was intentionally broad enough to retrieve phenotype-indirect fibromyalgia trials used only as contextual evidence. Supplementary discovery was used to improve recall but not to claim systematic completeness.

**Table A2:** Registered studies relevant to the evidence horizon.

Registry	Population/Phenotype	Design and Intervention	Outcome Relevance	Publication Status
NCT04678895 [41]	Painful diabetic neuropathy; phenotype-direct.	LDN compared with placebo in a crossover framework.	Direct placebo-anchored efficacy and tolerability in painful diabetic neuropathy.	No linked results publication identified in the updated search through 31 July 2026.
NCT05537935 [42]	Neuropathic pain in adults with HIV.	Twelve-week LDN study with pain and inflammatory-marker outcomes.	Direct clinical and biomarker evidence if phenotype ascertainment is adequately reported.	No linked results publication identified in the updated search through 31 July 2026.
NCT06723561 [43]	Central neuropathic pain after traumatic spinal cord injury.	Pilot treatment with LDN 4.5 mg/day for 12 weeks.	Feasibility and preliminary effect estimates in a direct central neuropathic phenotype.	No linked results publication identified in the updated search through 31 July 2026.
NCT06306157 [44]	Complex regional pain syndrome; mixed phenotype unless type II is separately characterized.	LDN compared with placebo with extended follow-up.	Placebo-controlled contextual evidence; phenotype-specific interpretation will depend on lesion ascertainment.	No linked results publication identified in the updated search through 31 July 2026.

Registry information and publication-status assessments are reported as of 31 July 2026 and may have changed subsequently.
